# Feasibility of a Digital Intervention to Promote Healthy Weight Management among Postpartum African American/Black Women

**DOI:** 10.3390/ijerph18042178

**Published:** 2021-02-23

**Authors:** Melissa A. Napolitano, Cherise B. Harrington, Loral Patchen, Lindsey P. Ellis, Tony Ma, Katie Chang, Azar Gaminian, Caitlin P. Bailey, W. Douglas Evans

**Affiliations:** 1 Department of Prevention and Community Health, Milken Institute School of Public Health, The George Washington University, Washington, DC 20052, USA; amgaminian@gwmail.gwu.edu (A.G.); cpbailey@gwu.edu (C.P.B.); wdevans@gwu.edu (W.D.E.); 2 Department of Exercise and Nutrition Sciences, Milken Institute School of Public Health, The George Washington University, Washington, DC 20052, USA; 3 Department of Public Health Education, North Carolina Central University, Durham, NC 27707, USA; cherise.harrington@nccu.edu; 4 Women’s and Infants’ Services, Medstar Washington Hospital Center, Washington, DC 20010, USA; Loral.Patchen@medstar.net (L.P.); Lindsey.P.Ellis@medstar.net (L.P.E.); 5 Benten Technologies, Manassas, VA 20110, USA; TonyMa@bententech.com (T.M.); hkchang@bententech.com (K.C.); 6 Department of Global Health, Milken Institute School of Public Health, The George Washington University, Washington, DC 20052, USA

**Keywords:** postpartum, weight loss, diet, activity behaviors, digital intervention

## Abstract

The study aim was to implement and evaluate the feasibility of a culturally informed (“BeFAB”) app for African American/Black women to address postpartum weight. Women (*n* = 136; mean age = 27.8 ± 5.4; mean BMI = 32.5 ± 4.3) were recruited from postpartum units, and randomly assigned to receive BeFAB (*n* = 65) or usual care (*n* = 71) for 12 weeks. App content included didactic lessons delivered via a virtual coach, app-based messages, goal setting and tracking, and edutainment videos. Feasibility outcomes included recruitment, retention and engagement, and self-reported acceptability. Behavioral (i.e., diet, physical activity), psychosocial (i.e., stress, coping, support, self-efficacy) and weight outcomes were also examined. Recruitment goals were met, but attrition was high, with 56% retention at 12 weeks. Approximately half of participants accessed the app and set a goal ≥one time, but <10% reported achieving a nutrition or activity goal. Among study completers, ≥60% found the app content at least somewhat helpful. Within-group changes for BeFAB among completers were found for increased moderate-to-vigorous physical activity and decreased fruit/vegetable intake and weight. Findings indicate initial feasibility of recruiting postpartum women to participate in a digital healthy body weight program but limited use, reflecting low acceptability and challenges in engagement and retention. Future research is needed on strategies to engage and retain participants in postpartum interventions.

## 1. Introduction

Approximately 40% of women of childbearing age have obesity, with an even higher prevalence among African American/Black (AA/Black) women [1]. Obesity comorbidities represent major leading causes of preventable death and lost quality of life in the United States [2] and worldwide [3]. The health consequences of obesity during preconception and pregnancy are significant, including increased risk of diabetes and chronic hypertension [4]. These long-term health risks are exacerbated by excess weight retention following delivery. AA/Black mothers are particularly at risk of experiencing excess postpartum weight retention [5], pointing to a need for culturally relevant interventions during this critical window in the lifecycle of both mother and child.

Studies to address maternal weight have focused on the prevention of excess gestational weight gain and postpartum weight loss. Intervention timing and strategies have varied, including a focus on gestational weight gain, healthy eating, and goal setting during pregnancy [6,7,8,9], postpartum weight loss [10,11,12,13,14,15,16,17,18], a combination [19], and a direct comparison [20]. The postpartum period represents a teachable window for intervention as women may be motivated to prevent future disease prevention for themselves and their families. For example, a two-year follow up of a randomized controlled trial (RCT) for postpartum weight loss reported that, among women who did not have a second pregnancy during the follow-up period, significant weight loss was maintained two years post-intervention [11]. Characteristics of effective postpartum weight loss interventions were summarized in a systematic review and meta-analysis [21], with more effective interventions being delivered by health professionals including a combination of both diet and exercise goals. Despite disparities in overweight/obesity prevalence [1] and maternal child health [22] outcomes by race, only two of the postpartum weight loss studies cited above directly addressed the needs of AA/Black women. This lack of attention may be due to the difficulties associated with reaching this population and engaging them in the research process.

Digital platforms are an effective means of delivering behavioral interventions and show promise for reaching hard-to-reach populations, such as racial/ethnic minorities [23,24] who show high smartphone usage and internet engagement [25]. Digital interventions also provide opportunities for participant co-creation of content (i.e., content co-generated by users and investigators), real-time monitoring of participant engagement, and the ability to reach more individuals using fewer resources than traditional in-person interventions. Taken together, digital interventions represent a promising method for reaching AA/Black women postpartum, when behavioral weight loss interventions can have a critical impact on the health of both mother and family [26].

This article describes the feasibility of implementing a digital healthy body weight intervention designed for postpartum AA/Black women: BeFAB (Be Fabulous After Baby, with a dual meaning of Be Fit After Baby) [27]. Feasibility metrics include recruitment, retention and engagement, and acceptability. Weight, behavior and psychosocial factors (i.e., stress, coping, support, self-efficacy, diet, and physical activity) were exploratory outcomes based on a model in which a culturally relevant intervention would lead to engagement with the intervention and would be related to changes in Healthy Eating and Activity Lifestyle (HEAL) behaviors and weight through improved coping skills and lowered stress, or increased self-efficacy. The BeFAB intervention was conceptualized to examine those two pathways through coping skills and lowered stress or though efficacy expectations and support.

## 2. Materials and Methods

### 2.1. Study Design

This study was a 3 month feasibility study to examine the implementation of a digital healthy body weight program for postpartum AA/Black women (*n* = 136). The sample size was determined based on detecting a mean weight loss difference of 4.45 kg between the groups, and assumed an attrition rate of 20% [27]. This study was approved by the Institutional Review Board at The George Washington University (#091732), with an Interagency Agreement with MedStar Hospital Center. Participants provided written informed consent.

### 2.2. Participants

Participants were recruited from the inpatient postpartum units within the first three days after giving birth. Recruitment occurred from May 2019 to November 2019. Inclusion criteria included self-identification as African American or Black, aged 18–40 years old, BMI 25–40, available for a 12 week self-administered online program and willing to complete end-of-study surveys online, no current contraindications for physical activity, own a smartphone or tablet, and Facebook user. Exclusion criteria were: current/planned use of weight loss medications (including over the counter) or other structured weight loss strategies, major psychiatric diagnosis not stable for at least 6 months, any medical or psychological condition that would make weight loss/physical activity/diet modification unsafe or unwise. See Table 1 for demographic characteristics of the sample.

### 2.3. Procedures

**Figure 1:**
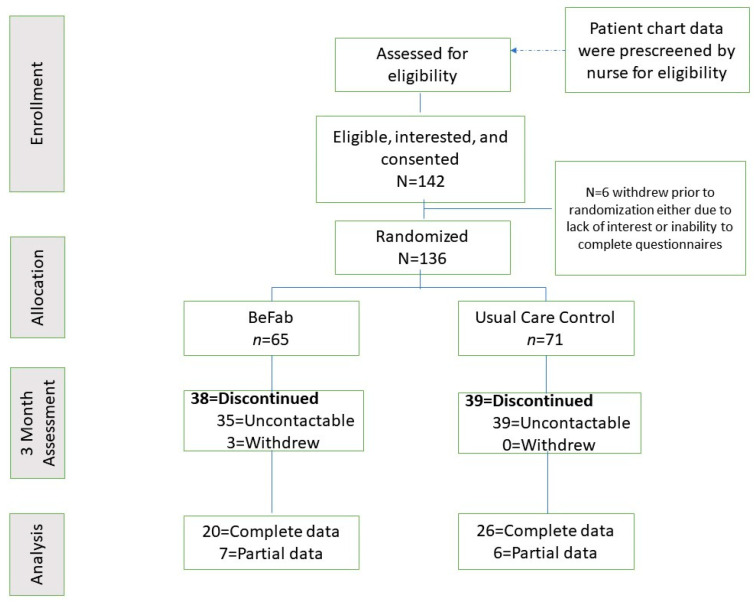
Enrollment flow diagram.

Participants were approached and informed about the opportunity to participate in a research study. Following initial eligibility screening by a member of the clinical team, the clinical research coordinator guided the participant through the consent and enrollment process, which included describing this study in detail, answering questions, and obtaining informed consent. All participants completed the Physical Activity Readiness Questionnaire (PAR-Q) [28], with any affirmative responses requiring further screening and approval prior to study enrollment. Demographic and anthropometric information was obtained via the patient chart. Participants completed their baseline questionnaires online via REDCap and were then randomly assigned to one of the two study groups (BeFAB or Usual Care) using the Randomization function in REDCap. See Figure 1 for the enrollment flow diagram.

### 2.4. Interventions

#### 2.4.1. BeFAB (*n* = 65)

**Figure 2:**
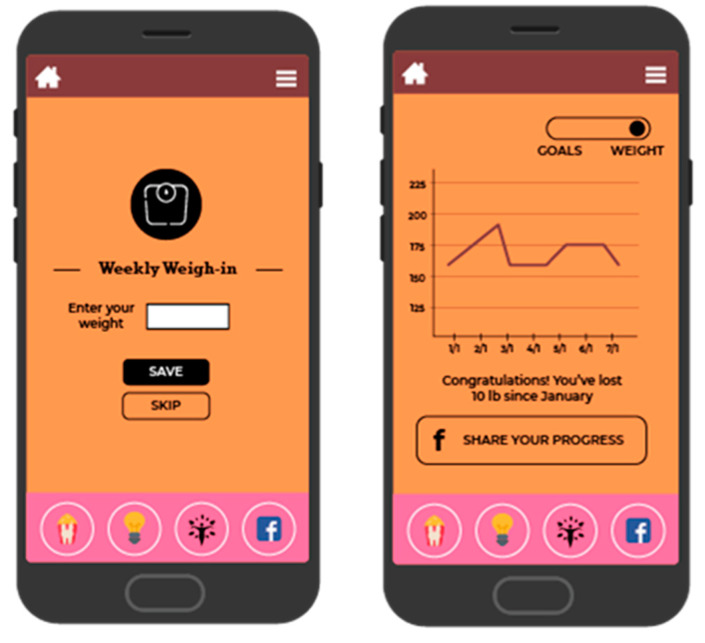
Screenshot of weight tracking and feedback.

**Figure 3:**
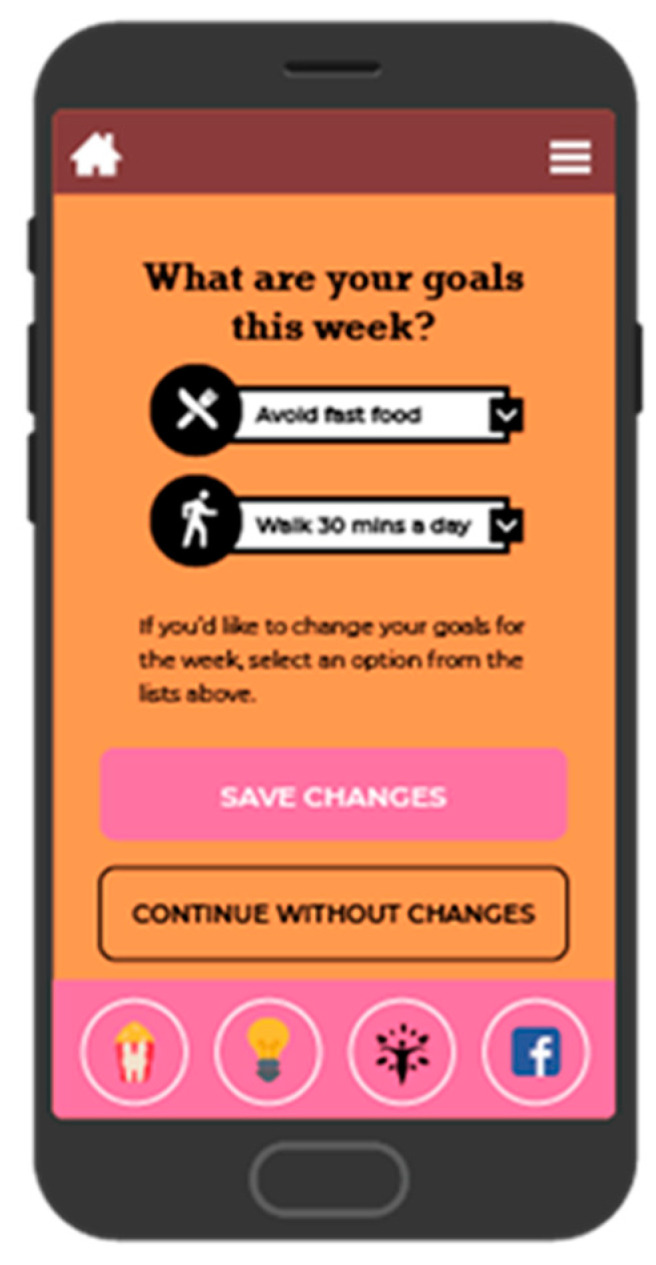
Screenshot of goal setting.

**Figure 4:**
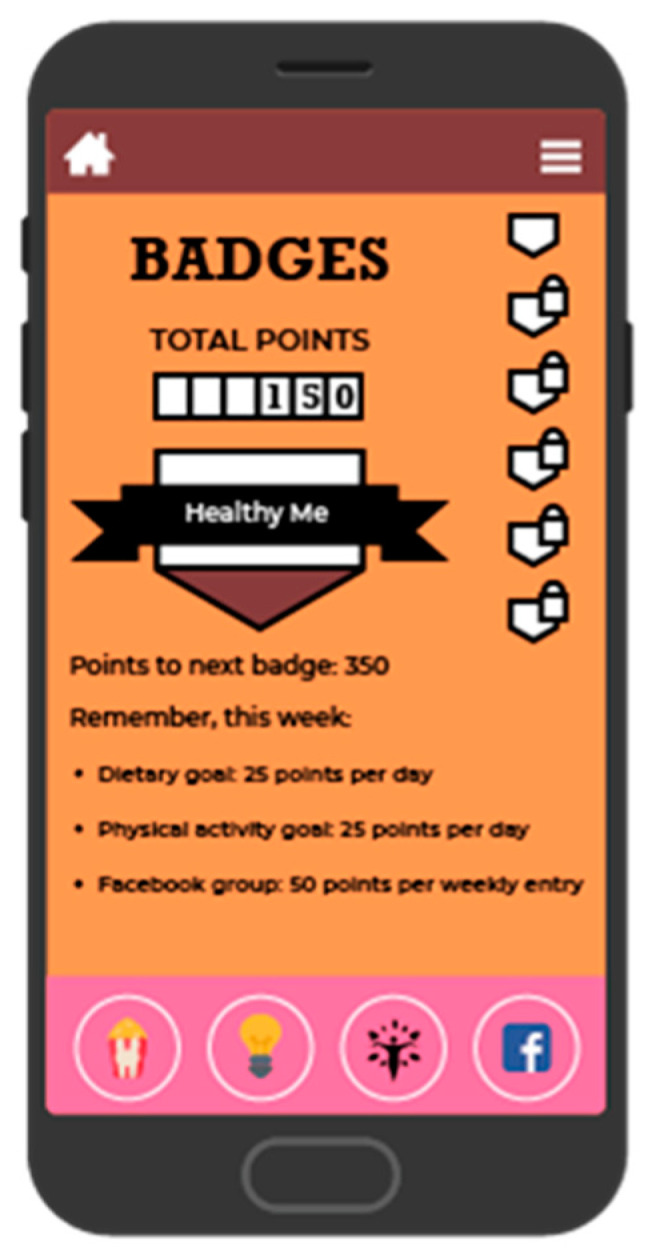
Screenshot of badge and point system.

BeFAB consisted of the culturally specific, branded BeFAB app developed by the research team integrated with a private Facebook group. Upon randomization, participants were instructed to download the app and were invited to join the private Facebook group. Participants received 12 weeks of content adapted from the Diabetes Prevention Program [29]. Lesson topics were tailored to the social and familial context of having a new baby along with formative work identifying key social, cultural and environmental factors. A community advisory board was convened providing additional input and feedback on the lessons and proposed focus (for more detail, see Evans et al. [27]). Sample content topics included “Tip the Calorie Balance”, “Being Active with Family”, and “Stress, Family, and Weight”. The content was delivered via didactic lessons, specifically “Dr. C’s Coaching Corner” and edutainment videos of the “BeFAB” Ladies. In-app messages were delivered five days per week on topics such as making over meals, managing cravings, and tips on how to be creative and fit in physical activity. Participants could track their weight and receive feedback (Figure 2), and also had the ability to choose one of six nutrition goals weekly (e.g., “Limit sugary drinks like juice and soda to no more than 1 per day, Limit junk and high fat foods to no more than 1 per day”) and one of six physical activity goals weekly (e.g., “Do 30 min of physical activity like walking or working around the house, Watch less than 2 h of TV per day”) and monitor their progress on those goals (see Figure 3; for more detail, see Evans et al. [27]). Participants received virtual “badges” such as the “Healthy Me” badge for meeting self-monitoring milestones (see Figure 4). Facebook posts were designed to create a sense of community and address neighborhood and social environment factors (e.g., “How to select healthy options when out with friends? Try some of these options: Drink water or unsweetened tea, share a main dish, pass on the buffet, select the side dishes. Share what has worked for you.”).

#### 2.4.2. Usual Care Control (*n* = 71)

All participants received the usual care as a patient. This included the provision of written information regarding resumption of physical activity postpartum, weight-related expectations and return to pre-pregnancy weight, healthy eating, physical activity guidance and suggestion to monitor weight.

### 2.5. Treatment Fidelity and Engagement

Within a week of being randomized, all participants received a follow-up text message and/or email from research staff to verify participant email address to send a $10 e-gift card incentive for completing the online survey. To boost app engagement, BeFAB group participants began receiving a weekly text from study staff once a week with encouraging messages and reminders to watch videos or update weekly goals, (e.g., “Have a great start to the week by setting new nutrition or physical activity goals! Check out what the BeFAB ladies are up to this week for ideas!”).

When participants from either study group reached 12 weeks from their randomization date (the month 3 [M3] follow-up timepoint), an automated email was sent from REDCap [30] with a personalized link to the end-of-study survey. Additionally, participants received a similar text message from study staff with a link to the end-of-study survey. Automated emails and text message attempts continued to be sent once a week to participants until the end-of-study survey was completed or 8 weeks passed from their first notice. The incentive for completing the end-of-study survey was a $10 e-gift card. To encourage end-of-study survey completion, a raffle was introduced for the chance to win 1 of 14 $50 e-gift cards. Raffles were announced by text message along with their link to the survey. Participants who completed the survey were automatically entered and had up to 2 chances to win an e-gift card. Raffles were held once a month with 2 winners each month who were notified by text message.

### 2.6. Measures

#### 2.6.1. Weight

At baseline, weight was extracted from the documented weight in the patient’s chart entered on admission to labor and delivery, which is based on patient self-report. At the M3 follow up, patients were also asked to self-report their weight.

#### 2.6.2. Activity Behaviors

Questions about physical activity included those from the International Physical Activity Questionnaire (IPAQ) on moderate and vigorous-intensity activity [31]. An additional question was asked about family time activity: “How many times per month does your family do some kind of physical activity together, such as dancing, walking, playing sports, or going to the park?” [32]. Sedentary time was assessed by asking participants, “On an average day, how many hours a day do you watch TV?” Physical activity data were cleaned and scored according to standard protocols with bouts of greater than 180 min truncated to equal 180 min [33], from which weekly averages of moderate-intensity (MPA), vigorous-intensity (VPA) and moderate-to-vigorous-intensity minutes of activity were calculated. Family-time physical activity responses were truncated to no more than 30 times per month; TV hours were truncated to no more than 16 h per day.

#### 2.6.3. Dietary Behaviors

Questions about dietary behaviors included questions adapted from the National Health and Nutrition Examination Survey [34]. The following items related to fruit and vegetable consumption were summed and divided by 7 to report an average number of servings per day: “How often do you drink fruit juices such as orange, grapefruit, or tomato;” “Not counting juice, how often do you eat fruit; “How often do you eat green salads (per week);” “Not counting salads, how many servings of vegetables do you usually eat? (Example: A serving of vegetables at both lunch and dinner would count as 2 servings).” Fast-food consumption consisted of the item “On how many of the past 7 days did you eat food from a fast-food restaurant, such as McDonald’s, KFC, Pizza Hut, Taco Bell, or a local fast-food restaurant?” [32,34] and was reported in times per week.

#### 2.6.4. Self-Efficacy and Social Support

Exercise self-efficacy (ESE) [35]. The ESE is a 5-item questionnaire assessing one’s confidence to be active when faced with 5 common barriers (e.g., bad weather, lack of time). Items were summed to create a composite score (range 5–25).

Weight self-efficacy (WEL) [36]. The WEL measures perceived control over eating behaviors and incorporates 20 different eating-related situations (e.g., social events, distractions, emotions). Items were summed to create a composite score (range 20–180).

Social support. Information and emotional support derived from the group were measured with two subscales (each 8 items) [37] assessing support from an online Facebook community: (1) emotional support (e.g., “I gain a feeling of acceptance from using this group”); (2) informational support (e.g., I find valuable information from this group”). Reponses ranged from 1 = strongly disagree to 5 = strongly agree.

#### 2.6.5. Stress and Coping

The Rhode Island Stress and Coping Inventory (RISC) [38] was used to measure mothers’ stress levels and adopted coping strategies in relation to having a newborn baby. Items are assessed on a 5-point Likert scale, with 7 items focused on stress (e.g., “I felt I had more stress than usual”) and 5 items focused on coping (e.g., “I successfully solved problems that came up”). Stress and coping items were separately summed to create composite scores (stress: range 7–35; coping: range 5–25).

#### 2.6.6. Acceptability

Questions adapted from Napolitano et al. [39] were used to assess the acceptability of the intervention, including helpfulness and perceived engagement with the digital intervention and content.

#### 2.6.7. Engagement

Usage data were automatically uploaded to our technology partner, Benten Technologies, when a participant logged in. Benten Technologies maintained a database and provided it to the investigative team for analysis at the end of this study.

#### 2.6.8. Data Analysis

Recruitment, retention and engagement. We report descriptive information on recruitment and retention. Descriptive statistics are provided on participant engagement with the intervention (i.e., downloaded app usage metrics data).

Acceptability. Additionally, we analyzed both self-reported exposure to the content and acceptability of app features.

Behavioral, psychosocial, and weight outcomes. Given the high attrition and the feasibility nature of this study, only those participants with baseline and M3 data were included. To compare those with follow-up data to those who did not complete the M3 follow up, we also examined baseline demographic characteristics using *t*-tests for continuous variables and chi-squared tests for categorical variables.

To test intervention effects on these outcomes, we examined changes both within and between groups. For each outcome, Shapiro–Wilk tests were used to examine distributional normality. Normally distributed variables were tested using paired (within group) and unpaired (between-group) *t*-tests. Wilcoxon tests were used to test variables that were not normally distributed. All data were analyzed using RStudio Version 1.3.1056.

## 3. Results

### 3.1. Recruitment

The recruitment goal of 136 participants was met within a 7 month recruitment window. See Figure 1.

### 3.2. Retention and Engagement

Retention. Fifty-seven percent of the sample was lost to follow up (38 in BeFAB and 39 in Usual Care). There are no significant differences between those with follow-up data (*n* = 59) and those without (*n* = 77). See Figure 1 for retention information.

Engagement. Engagement results were downloaded directly from the technology partner. Fifty four percent of intervention participants accessed the BeFAB app and set a goal at least one time; however, less than 10% reported achieving either a nutrition or activity goal according to self-report. Additional metrics and *n* are reported in Table 2 below.

### 3.3. Acceptability

Acceptability metrics are available from completers only. The majority of those with complete data self-reported watching the video content, including the BeFAB edutainment videos (61%), and didactic lessons (63%). The majority rated these as either very or somewhat helpful (70% for entertainment and 65% for didactic) and found each type of video to be very or somewhat engaging (70% for entertainment and 65% for didactic).

### 3.4. Behavioral and Psychosocial Outcomes

Behavioral and psychosocial outcomes (i.e., stress, self-efficacy, diet, and physical activity) were assessed via a self-reported questionnaire. See Table 3 for results from baseline to M3.

#### 3.4.1. Activity Behaviors

There was a significant increase in weekly minutes of moderate-to-vigorous physical activity (338.5 min; *p* = 0.004)) and vigorous physical activity (228.2 min; *p* = 0.001) among the BeFab participants. Differences between the BeFab and Control groups were not significant. No other activity outcomes were found to be statistically significant from baseline to M3, although there was a trend for weekly vigorous physical activity also to increase among the control group (*p* = 0.05).

#### 3.4.2. Diet

BeFab participants reported, on average, 1.2 fewer servings per day of fruits and vegetables from baseline to M3 (*p* = 0.01) compared with 0.3 more servings per day by control participants (*p* = NS); differences between the groups were significant (*p* = 0.02). No significant between- or within-group differences were found for *fast food consumption*.

#### 3.4.3. Self-Efficacy, Social Support

No significant between- or within-group differences were found for *Exercise self-efficacy, Weight self-efficacy or Perceptions of support* from the Facebook group.

#### 3.4.4. Stress and Coping

No significant between- or within-group differences were found for *Stress* or *Coping*.

### 3.5. Weight

Weight loss from baseline to M3 among BeFAB participants was 12.6 kg (*p* < 0.001), compared with 8.3 kg among the Control participants (*p* < 0.001); differences between the groups were not significant.

## 4. Discussion

Postpartum weight retention is a significant public health concern. Results from this feasibility study indicate success in recruiting women in the postpartum period to participant in a program to address healthy eating and activity behaviors. The recruitment target of 136 was successfully attained within a 7 month timeframe, averaging an enrollment of approximately 19 participants per month, which is higher than similar weight management trials with postpartum women [12,14]. We attribute this success to a number of factors including the integration of the research team with the clinical providers, streamlining the enrollment process through recruiting postpartum women from postpartum units prior to discharge, enrollment by a member of the research team who is also a health care provider, and intervening at a critical window for intervention. To mitigate perceptions of coercion and to standardize recruitment, a written protocol was followed when approaching the patient on the postpartum unit. While successful, the feasibility of recruiting women on the postpartum unit also introduced some unexpected challenges. For example, one enrollment challenge was that to access the BeFAB app, a charged phone, accessible password, and sufficient memory were needed. The enrollment process was lengthy, and some potential participants were tired, did not wish to continue with the process, or were sleeping and could not be disturbed. Extending the enrollment period to prior to delivery could help mitigate the multiple demands of the immediate postpartum period.

While use of the BeFAB app was low, among those who completed the program, women found it to be acceptable with the majority reporting the BeFAB app content to be at least somewhat helpful. Acceptability was similar across content types, suggesting that women found the information provided in the didactic lessons as well as the edutainment videos useful for their healthy eating and physical activity goals. These findings are similar to others (e.g., [40,41]) and indicate that future studies should explore multiple ways of presenting and modeling relevant information. Informational and emotional support that women derived from the Facebook group was modest, which may indicate the nature of the postpartum period and participating in a group with unknown social network contacts.

When markers of engagement via app metrics were examined, approximately half of the BeFAB participants accessed the app at least once and set a goal, but less than 10% reported achieving *either* a nutrition or physical activity goal. It is unclear whether women were achieving the goals and not monitoring their progress in the app, or if they were unable to reach the target. It is also unknown whether the goals set were realistic. In our clinical experience, women have tended to set goals that were unachievable or unrealistic (e.g., achieving pregnancy weight by 6 weeks following significant gestational weight gain) with small incremental goals seeming to be less motivating. The badges did not appear to provide sufficient extrinsic motivation to encourage either goal achievement or monitoring within the app. The behavioral goals were based on previous work [41,42], varied in terms of intensity and difficulty, and provided women with the opportunity to change the goals each week. We did not obtain feedback on the goals, so we cannot determine whether they were realistic or too burdensome given stress and having a new baby at home. These results also suggest that additional methods of engaging women early in the study content, perhaps during pregnancy, to mitigate some of these challenges may be useful. We recommend that future digital interventions such as BeFAB use enhanced incentive systems, including contests that include both monetary and non-monetary rewards, and create support systems based on friendly competition. Following our conceptual model, we believe this kind of support system will increase engagement and lead to improved behavioral and healthy weight outcomes. We also recommend creating more elaborated goals that promote self-efficacy and increase a sense of accomplishment in the program, providing further reinforcement. This would involve extending the BeFAB model to a longer timeframe, increasing the didactic and narrative content to address additional goal setting, and promoting long-term maintenance of behavior change.

Despite engagement strategies used, more than 50% of the sample was lost to follow up, which was higher than anticipated. In comparison, 40% of women do not attend a postpartum clinical visit [43]. However, there were no demographic differences between completers versus non-completers. While attrition was high, it is recognized that the demands on mothers are also high during this period, with many competing priorities: baby care and care of other children, fatigue, general lack of time and attention available for self-care. There may be other factors related to attrition on our study, as well as not receiving postpartum care. The needs of mothers after childbirth have been summarized as: (1) informational; (2) psychological and practical (e.g., household chores) support; and (3) social connections through sharing experiences [44]. While our program addressed some of the above by providing information, skills training for coping with stress and ability to connect with others through social media, the practical demands (e.g., household, care) may have outweighed the other needs. Attrition may be related to the study design: our study recruited from the postpartum units prior to discharge. Women may have been initially motivated and excited but faced many challenges as noted above. Overcoming barriers to retention and finding relevant incentives for this population is a significant need to explore in future studies. Examining the timing of recruitment and intervention also will be an important factor for future studies. For example, recruitment prenatally, and inclusion of prenatal nutritional guidance to engage women before delivery, might assist with retention efforts. Other suggestions include using technology for communication (e.g., text messaging), finding ways to reduce burden such as home or virtual visits, and use of sufficient financial incentives [45].

Analyses were conducted among those with follow-up data only and specifically focused on those behavioral targets of the intervention: diet and physical activity, stress, support, and coping. BeFAB participants reported more moderate-to-vigorous physical activity at M3, with vigorous physical activity appearing to be related to this change. It is possible that vigorous physical activity is easier to recall as it tends to occur in discrete bouts compared with moderate-intensity or lifestyle activity [46]. More information is needed to better understand the type and nature of activities that are favored by postpartum women. It also would be important to have a validation on reporting of activity intensity as it may be possible women felt like the activity should fall into a vigorous category based on perceived exertion [47]. Despite these within-group difference, no between-group differences were found for the activity behaviors, likely due to the small sample sizes and variability in activity outcomes.

BeFAB participants reported approximately one serving fewer fruits/vegetables at M3, which was significantly different than those in the usual care condition. It is possible that the didactic information provided by BeFAB on fruit and vegetable serving sizes and/or serving goals may have resulted in women being more accurate in their reporting. In other words, reduced fruit/vegetable intake among the intervention might reflect greater knowledge of how much a serving of fruits and vegetables is rather than an actual decrease in consumption. This would simultaneously explain why an effect was not seen among the control group. Additionally, BeFAB did not provide a dietary target for the number of fruits and vegetables per day but rather focused on the MyPlate recommendation of filling one’s plate with vegetables. It may be that women were not clear on the overall dietary goals to target. Finally, the dietary screener questions were adapted from NHANES and are not perfectly aligned with the NHANES questions. For example, they do not parse out 100% fruit juice from sweetened fruit drinks and may not reflect a true dietary picture.

There were no significant between and within-group differences in stress or coping strategies. Only one session focused on stress and managing stress and may not have provided enough of dose for short term changes in these measures. Additionally, women have many demands and stressors with the arrival of a new baby. It may be difficult for women to implement the strategies during the 12 week timeframe of this study with these other multiple demands.

Finally, unsurprisingly in the postpartum period, both groups did lose significant weight from baseline, and BeFAB participants reported greater weight loss, on average, than control participants. However, these between-group differences were not significant, which could be due to the greater variability of weight loss outcomes among intervention versus control participants.

Although there are some promising results regarding recruitment and acceptability, there are a number of limitations. First, attrition was high and more than projected for the power calculations. Results should be interpreted cautiously as we were examining multiple outcomes on a small subset of completers only. Second, we did not have measured weight on participants. At baseline, predelivery weights were extracted from the patient charts which were self-reported values entered on admission to labor and delivery, which have been shown to have high concordance with measured weights [48]. The weight change data presented are thus based on baseline weights collected prior to delivery. We had anticipated timing the intervention with postpartum clinic visits such that weights could be gathered from the patient records at follow up. However, many of the women received postpartum care through satellite clinics which were not linked to the hospital records and the timing of the follow up did not match sufficiently to link to a 12 week outcome. Therefore, we asked women to self-report their weight at follow up which was a similar procedure their clinic team used for the predelivery weight. Ninety-eight percent of women reported this was from their last doctor’s visit or own scale. We did not have the resources to provide a scale, but future studies should consider scale provision to enhance self-weighing and outcome data collection. We do not have data on gestational weight gain and cannot compare the groups on this variable. We do not have data on the mode of delivery, which could relate to physical activity participation. However, all women completed a readiness questionnaire for physical activity as a screening tool for contraindications for physical activity. The physical activity measure was self-report and may have resulted in self-reporting bias. Similarly, we had included an adapted version of the NHANES dietary screener and were instructed by the clinical team that the measure was too long and some items were ultimately removed; thus we do not have data on outcomes such as sugar-sweetened beverage consumption. We did not collect data on breastfeeding status, employment status or length of maternity leave which could relate to many of the variables presented in this paper. Additionally, while the app was easily downloaded to an iPhone, the interface to an Android was more complicated. As a result, recruitment was eventually limited to only iPhone users, which may limit the generalizability of the findings. We do not know whether retention and engagement were related to connectivity or user-interface issues with the BeFAB app. There were several benefits derived from using an app that was synced to a user’s phone; however, future studies should explore technologies that can be used across devices. We also did not collect ongoing Facebook user data such as the number of posts seen or liked, or overt posts made to the group. These are important metrics to examine in future studies.

## 5. Conclusions

Study results indicate the feasibility of recruiting postpartum AA/Black women to participate in a program to address healthy eating and physical activity. BeFAB participants demonstrated increases in moderate-to-vigorous physical activity, vigorous physical activity alone, and decreases in weight from baseline to M3. These findings should be interpreted with caution given that the use of the app was low and attrition was high. Despite these limitations, this study presents some evidence that the tested intervention can have a positive effect but this needs to be investigated further in a large-scale trial. Given the needs of women in the postpartum period, interventions such as this one are difficult to implement and may need to include factors such as psychological support, story sharing, and strategies to assist in practical support. Future studies should continue to address the needs of women during the postpartum period to enable them to address their diet, physical activity, and weight for the lifelong health of themselves and their families.

## Figures and Tables

**Table 1:** Descriptive statistics.

	All Participants (*n* = 136)	Intervention (*n* = 65)	Control (*n* = 71)
Age (years)	27.8 ± 5.4	28.2 8 ± 5.6	27.5 8 ± 5.2
Weight (kg)	86.9 ± 14.1	86.4 ± 13.7	87.4 ± 14.5
BMI (kg/m^2^)	32.5 ± 4.3	32.1 ± 4.0	32.7 ± 4.6
% Non-Hispanic	91.2	89.2	93.0
Marital Status (%)			
Single, never married	78.5	78.1	78.9
Married or partnership	21.5	21.9	21.1
Childbearing History (%)			
Primiparous	22.1	20.0	23.9
Multiparous	76.5	80.0	73.2
Health Insurance (%)			
Medicaid	58.1	49.2	66.2
Medicare	1.0	0	1.4
Other	39.7	47.7	32.4

Note: Marital status *n* = 135 (intervention *n* = 64). Baseline weight and BMI refer to the documented weight in the patient’s chart entered on admission to labor and delivery, which is based on patient self-report.

**Table 2:** Engagement metrics downloaded from the BeFAB app.

	*n*	% of Randomized
Accessed the app at least one week	35	54%
Recorded weight at least once	9	14%
Watched at least one video	15	23%
Set goals for at least one week	32	49%
Achieved at least one activity goal	5	8%
Achieved at least one nutritional goal	4	6%

**Table 3:** Change in outcomes from baseline to post-intervention.

	Intervention		*p*-Value	Control		*p*-Value	Difference Scores ^b^ for Intervention vs. Control		*p*-Value
	Baseline Mean	M3 Mean		Baseline Mean	M3 Mean		INT (CI)	CON (CI)	
Physical Activity ^a^									
Weekly MVPA (min.)	*n* = 22		0.004	*n* = 19		0.41	338.5 (109.5, 567.6)	81.1 (−136.8, 299.0)	0.10
	279.9	618.4		364.2	445.3				
Weekly MPA (min.)	*n* = 22		0.19	*n* = 19		0.76	110.4 (−74.9, 295.7)	0.4 (−154.7, 155.5)	0.35
	180.0	290.1		229.2	229.6				
Weekly VPA (min.)	*n* = 22		0.001	*n* = 19		0.05	228.2 (80.6, 375.8)	80.7(−18.2, 179.6)	0.09
	100.1	328.3		135.0	215.7				
Family-time PA (times per month)	*n* = 24		0.07	*n* = 30		0.76	−4.1 (−8.1, −0.2)	−1.1 (−5.6, 3.4)	0.30
	10.4	6.3		9.3	8.2				
TV watching (hours per day)	*n* = 23		0.82	*n* = 28		0.64	0.5 (−0.7, 1.8)	−0.2 (−0.9, 0.5)	0.28
	2.7	3.3		2.2	2.0				
Dietary Behaviors ^a^									
Fruits and vegetables (servings per day)	*n* = 22		0.01	*n* = 26		0.59	−1.2 (−2.1, −0.3)	0.3 (−0.6, 1.3)	0.02
	3.5	2.3		3.2	3.5				
Fast-food consumption (times per week)	*n* = 22		0.40	*n* = 26		0.54	0.2 (−0.6, 0.9)	−0.1 (−0.9, 0.7)	0.57
	2.1	2.3		2.8	2.7				
Self-Efficacy									
Exercise self-efficacy ^b^	*n* = 26		0.73	*n* = 30		0.52	0.4 (−2.0, 2.9)	0.6 (−1.2, 2.3)	0.92
	13.7	14.2		14.4	15.0				
Weight self-efficacy ^a^	*n* = 23		0.06	*n* = 27		0.69	15.1 (−2.6, 32.7)	−2.9 (−21.7, 16.0)	0.16
	126.9	142.0		138.5	135.6				
Stress and Coping ^a^									
Stress score	*n* = 21		0.93	*n* = 26		0.12	0.2 (−3.4, 3.8)	1.7 (−0.4, 3.9)	0.45
	15.9	16.1		15.3	17.0				
Coping strategies score	*n* = 21		0.87	*n* = 26		0.98	0.8 (−1.9, 3.4)	−0.1 (−2.2, 2.0)	0.59
	18.7	19.4		18.1	18.0				
Weight (kg) ^a^	*n* = 27		<0.001	*n* = 30		<0.001	−12.6 (−20.0, −5.6)	−8.3 (−10.3, −6.2)	0.23
	86.4	74.2		87.4	78.7				

*Note*: Confidence interval, CI; moderate-to-vigorous-intensity physical activity, MVPA; moderate-intensity physical activity, MPA; vigorous-intensity physical activity, VPA; physical activity, PA. ^a^ Variables at baseline and M3 were not normally distributed, thus non-parametric tests were used. ^b^ Variables were normally distributed, thus parametric tests were used.

## Data Availability

Deidentified data that support the findings of this study will be made available 6 months following publication for 5 years to researchers submitting a request and data sharing agreement to the corresponding author. The study protocol is available elsewhere [27].
